# Density Functional Theory-Based Studies Predict Carbon Nanotubes as Effective Mycolactone Inhibitors

**DOI:** 10.3390/molecules27144440

**Published:** 2022-07-11

**Authors:** Nafiu Suleiman, Abu Yaya, Michael D. Wilson, Solomon Aryee, Samuel K. Kwofie

**Affiliations:** 1 Department of Materials Science and Engineering, University of Ghana, Accra LG 54, Ghana; nsuleiman@st.ug.edu.gh (N.S.); ayaya@ug.edu.gh (A.Y.); 2 Department of Parasitology, Noguchi Memorial Institute for Medical Research (NMIMR), College of Health Sciences (CHS), University of Ghana, Accra LG 581, Ghana; mwilson@noguchi.ug.edu.gh; 3 Department of Medicine, Loyola University Medical Center, Maywood, IL 60153, USA; 4 Department of Biomedical Engineering, School of Engineering Sciences, College of Basic and Applied Sciences, University of Ghana, Accra LG 77, Ghana; aryeeesolomon@gmail.com; 5 West African Centre for Cell Biology of Infectious Pathogens, Department of Biochemistry, Cell and Molecular Biology, College of Basic and Applied Sciences, University of Ghana, Accra LG 54, Ghana

**Keywords:** mycolactone, nanotubes, density functional theory (DFT), boron nitride nanotubes (BNNTs), carbon nanotubes (CNTs), HOMO–LUMO orbital, inhibitors

## Abstract

Fullerenes, boron nitride nanotubes (BNNTs), and carbon nanotubes (CNTs) have all been extensively explored for biomedical purposes. This work describes the use of BNNTs and CNTs as mycolactone inhibitors. Density functional theory (DFT) has been used to investigate the chemical properties and interaction mechanisms of mycolactone with armchair BNNTs (5,5) and armchair CNTs (5,5). By examining the optimized structure and interaction energy, the intermolecular interactions between mycolactone and nanotubes were investigated. The findings indicate that mycolactone can be physically adsorbed on armchair CNTs in a stable condition, implying that armchair CNTs can be potential inhibitors of mycolactone. According to DOS plots and HOMO–LUMO orbital studies, the electronic characteristics of pure CNTs are not modified following mycolactone adsorption on the nanotubes. Because of mycolactone’s large π-π interactions with CNTs, the estimated interaction energies indicate that mycolactone adsorption on CNTs is preferable to that on BNNTs. CNTs can be explored as potentially excellent inhibitors of mycolactone toxins in biological systems.

## 1. Introduction

Mycolactone is an immunosuppressive polyketide-derived macrolide toxin secreted by a group of very closely related pathogenic mycobacterial species, including *Mycobacterium ulcerans*, *Mycobacterium liflandii* (Frog pathogen), *Mycobacterium pseudoshottsii*, and some strains of *Mycobacterium marinum* (Fish pathogen) [1]. In 1999, George and colleagues succeeded in isolating and deciphering a cytotoxic component from *M. ulcerans* lipid extracts [2]. The name mycolactone was coined based on the mycobacterial origin and macrolactone structure of the toxin extract: a 12-membered lactone ring to which is appended a C5-O-linked polyunsaturated acyl side chain and a C-linked upper side chain comprising C12–C20 [3]. The structural characteristics of three mycolactones (A/B, C, and D) isolated from clinical isolates of *M. ulcerans* have been determined. In humans, mycolactone is known to be associated with the pathogenesis of Buruli ulcer (BU), a disease caused by *M. ulcerans* [4]. BU is one of the most neglected tropical diseases. However, it is the third most common disease caused by a *mycobacterium*, besides tuberculosis and leprosy [5]. Because mycolactone is lipophilic, it is thought to passively penetrate cell membranes and bind to crucial proteins involved in platelet and mast cell exocytosis, hence slowing wound healing [6,7]. According to recent research, mycolactone exerts a pleiotropic effect and disrupts basic cellular processes such as cell attachment, signaling pathways, cell proliferation, cell death, and inflammation [8]. This accounts for the painless, slow-healing ulcer lesions associated with BU. However, significant progress has been made in understanding the multifaceted role of mycolactone in host colonization. Some of these roles include an immunomodulatory effect, targeting the Sec61 channel and facilitating immunological escape [8], and an analgesic effect, targeting AT2R receptors and accounting for painlessness in individuals with early BU lesions [3]. Despite this progress, there remain some challenges since generated neutralizing antibodies have the drawback of being produced from hybridomas [9].

Biomaterials, in recent times, have become essential elements that are used in a wide range of biomedical and clinical applications. The employment of nanoparticles in these fields has a lot of promise, owing to the high ratio of surface atoms, which changes the physicochemical characteristics and boosts chemical reactivity. Nanotubes are divided into two categories comprising single-walled nanotubes (SWNTs) and multiple-walled nanotubes (MWNTs) [10,11]. Nanotubes can be further classified into three configurations consisting of the armchair, zigzag, and chiral nanotubes. The difference in the various types of NTs is based on how the sheet is “rolled up” during its production process. The radius of the closing cylinder and the rolling axis relative to the hexagonal network of the nanosheet allow for different forms of SWNTs [12,13]. The outside diameter of single-walled CNTs is typically between 1 and 2 nm; however, the outer diameter of multi-walled CNTs can approach 100 nm. Increasing the number of layers in MWNTs always increases the number of flaws, making them easier to change and functionalize, usually at the expense of physical qualities of degradation [14].

Among them, CNTs have proven to be powerful tools for improved biomedical approaches in the management of numerous diseases, such as cancer and Alzheimer’s disease [15]. Carbon nanotubes (CNTs) are widely employed in biology and medicine for in vitro and in vivo detection, imaging, and drug administration [16]. They are one of the most splendid nanostructures. CNTs were also found to have low toxicity when used as a medication carrier [17]. CNT pores (CNTPs) have been discovered to have water-salt permselectivity values comparable to those of conventional desalination membranes [18] due to their great chemical stability. CNTs have an excellent ability to penetrate cell membranes, and their carbon atoms have sp2 hybridization, enabling their functionalization with almost every biomolecule or compound [15]. This allows them to target cells and deliver drugs under the appropriate environmental stimuli [15]. BNNTs have also been extensively used in many applications [19]. BNNTs—structural analogs of CNTs—have piqued the interest of researchers in nanomedicine due to their one-dimensional (1D) physical structure, stable chemical composition, low toxicity, and a plethora of other properties, making them particularly promising for drug carriers, tissue scaffolds, chemical agents for boron neutron capture therapy (BNCT), and irreversible electroporation for cancer therapy [20,21,22]. BNNTs are naturally noncytotoxic and can be functionalized with chemical groups to bind proteins and cells. According to research, BNNTs have considerable promise in biosensor and nanomedicine applications [23]. Both CNTs and BNNTs exhibit comparable heat conductivity and mechanical stiffness.

Few studies have been conducted seeking to annihilate the effect of mycolactone on host tissues. A study [9] developed an anti-mycolactone immune-sera and monoclonal antibody (mAb) showing in-vitro-neutralization activity by immunizing mice with a protein conjugate of a non-toxic synthetic truncated mycolactone variant. Despite this progress, there remain some challenges since the neutralizing antibodies have the drawback of being produced from hybridomas [9]. This renders them suboptimal for the treatment of human subjects owing to the risk of anti-murine antibody reactions [24]. Although, humanized variants of these murine antibodies can be obtained to possibly treat BU, this technique is time-consuming and does not necessarily ensure that the ‘converted’ antibodies will retain the same effectiveness or might still evoke an immunogenic response [24]. Again, the currently known antibodies against mycolactone are few, hence the need to investigate novel inhibitors for mycolactone to optimize BU treatment. The purpose of this paper was to evaluate if armchair BNNTs (5,5) and CNTs (5,5) can behave as inhibitors when DFT calculations are used in this investigation. The electrical properties of mycolactone, as well as its interactions with armchair BNNTs (5,5) and CNTs (5,5) were thoroughly explored. When compared to other NT topologies, the NT configuration (5,5) was shown to be the most stable following biomolecular adsorption [25].

## 2. Materials and Methods

### Computational Details

The original structures of the armchair BNNTs (5,5), armchair CNTs (5,5), and mycolactone molecules were modeled using the Avogadro [26] Following that, we optimized their molecular geometries using the DFT with the Quantum Espresso [27]. The hybrid B3LYP (Becke’s three-parameter hybrid functional with Lee-YangParr correlation functional B3LYP at a 6-31G* basis set was used for all quantum-chemical calculations. In the investigation of the electrical structure and characteristics of various BN nanostructures, B3LYP has been shown to be a reliable and widely used functional [28]. For the treatment of intermolecular interactions, it is generally accurate [29]. Several electronic parameters were estimated using the DFT approach with the hybrid B3LYP [30,31], including frontier molecular orbitals, gap energies, and reactivity descriptors. Quantum Espresso software was also used to generate density of state (DOS) plots. The cartesian coordinates are provided in Supplementary File S1.

## 3. Results and Discussion

### 3.1. Molecular Geometry and Adsorption Energy

The structures were optimized before the pure DFT calculations using the self-consistent density functional tight-binding method with empirical dispersion (SCCDFTB) [32], which is derived from the DFT total energy’s second-order expansion. The SCC-DFTB-D approach is two or three orders of magnitude faster than pure DFT, and it has been effectively employed in analyzing the electronic structures and properties of massive systems [33]. It also has lesser precision than pure DFT [34]. The pure DFT approach was then used to finely calculate the structures and properties of the interacting system.

**Figure 1:**
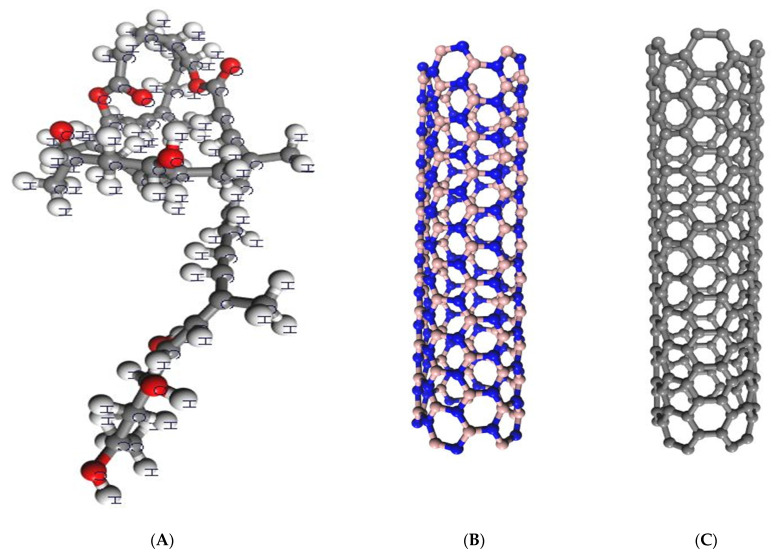
Optimized structures of mycolactone, armchair BNNT (5,5), and armchair CNT (5,5). (**A**) the optimized structure of mycolactone, (**B**) the structure of armchair BNNT (5,5), and (**C**) the structure of armchair CNT (5,5). Carbon, boron, and nitrogen are represented by grey, brown, and blue, respectively.

To optimize the structures of the mycolactone, armchair BNNT (5,5), and armchair CNT (5,5), the computationally fast SCC-DFTB-D approach was used. After that, the optimized geometries were employed as the starting points for pure DFT calculations. There were no constraints used, and frequency calculations were carried out to guarantee that the optimized structures were indeed at their local minimum. Because the SCC-DFTB-D approach is theoretically approximated by the pure DFT method, this two-step optimization procedure greatly accelerated our calculations while preserving precision. The SCC-DFTB-D approach proved to be a very useful tool for researching the properties of large systems. Figure 1 depicts the optimized structures as well as the atom labeling. According to the findings, the mycolactone developed a non-planar form. The average bond lengths of the B–N bonds in BNNT and the C–C bond length in CNT, as shown in the optimized geometries of the nanotubes in Figure 1B,C, are 1.47 and 1.43 Å, respectively, which agree well with earlier theoretical conclusions [35,36].

**Figure 2:**
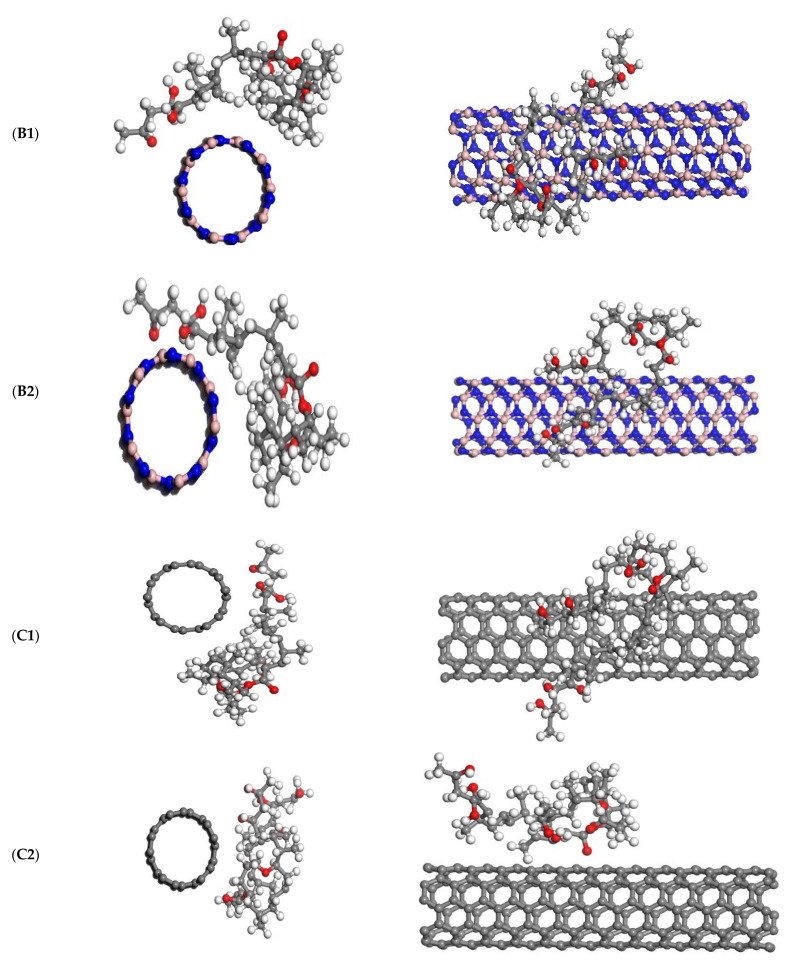
The top and side views of the optimized structures of the mycolactone on armchair BNNT (5,5) and armchair CNT (5,5). (**B1**)—Mycolactone on a center of BNNT’s surface, (**B2**)—Mycolactone on the far end of the BNNT’s surface, (**C1**)—Mycolactone on the center of the CNT’s surface, and (**C2**)—Mycolactone on the far end of the CNT’s surface.

The optimization of the geometry of the interacting complex structures was then used to investigate the adsorption of mycolactone on the outer surface and near the tube cave. To ensure that different sections of the mycolactone molecule interact correctly with the tube surface, it was initially placed in various positions in the tube and its orientation was changed. Figure 2 shows the optimized structures of mycolactone on armchair BNNT (5,5) and armchair CNT (5,5), as well as their relative orientations in top and side views. Table 1 shows the DFT (PBE) level of theory estimated binding energies and equilibrium bond lengths at the binding sites of mycolactone functionalized nanotube complexes. In Figure 2, the two most representative locations for the mycolactone on BNNT are labeled B1 and B2, respectively. The representative locations of C1 and C2 for mycolactone on CNT are also labeled in Figure 2. According to the optimized structures, the most stable adsorption sites for mycolactone on BNNT are at the tube’s two ends. The mycolactone molecule interacts weakly with the BNNT surface towards the tube’s ends in the B1 case (Figure 2), which has a maximum interaction energy of 43.90 kcal/mol.

**Figure 3:**
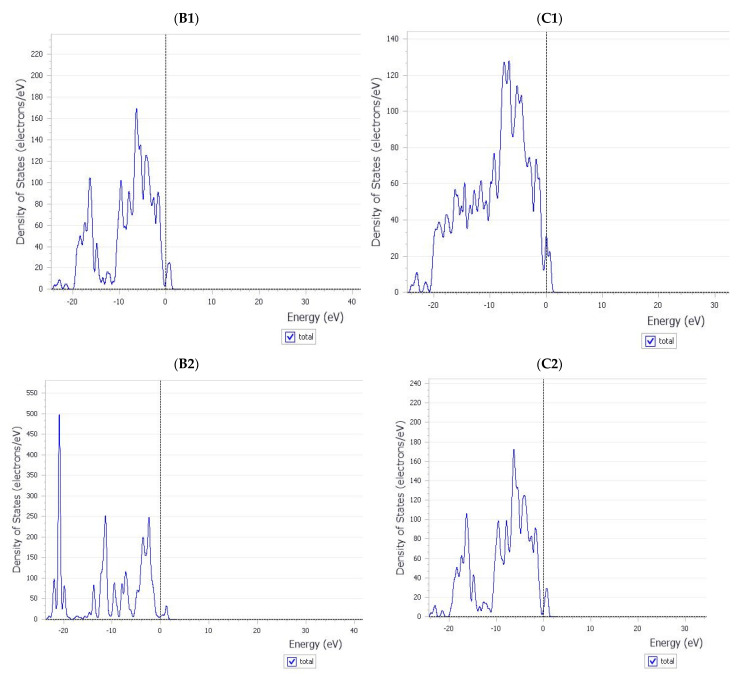
The density of states (DOS) of the mycolactone- nanotube complexes calculated at the DFT level. (**B1**)—Mycolactone on a center of BNNT’s surface, (**B2**)—Mycolactone on the far end of the BNNT’s surface, (**C1**)—Mycolactone on the center of the CNT’s surface, and (**C2**)—Mycolactone on the far end of the CNT’s surface.

The features of good inhibitors do not vary considerably during the inhibition process, allowing them to block the target. Following that, we investigated whether mycolactone adsorption affects the characteristics of nanotubes. At the DFT level, the density of states (DOS) of the complexes were determined and compared (Figure 3). In these figures, the HOMO energies are adjusted to zero. The fact that BNNTs are wide-gap semiconductors with a HOMO–LUMO gap of roughly 5 eV is widely known. The HOMO–LUMO gap of the armchair BNNTs (5,5) was predicted to be around 4 eV by our calculations at the DFT (PBE) level. This conclusion was consistent with DFT and tight-binding calculations based on LDA [37]. The CNTs’ DOS graphs show that there were no gaps between the HOMO and LUMO orbitals, indicating that they had electrical conductivity. The tight-binding band-structure calculations [38] verified this observation. The B1 and B2 examples had gaps of 0.70 and 0.83 eV, respectively. When compared to the gap of isolated BNNT, the interaction with the mycolactone molecule significantly altered the gap. The adsorption of mycolactone on the nanotubes introduced some new states around the LUMO areas, and the HOMO level was displaced to the lower regions, as shown in the images when compared to the pure BNNT. As a result, the BNNT tube gap was significantly reduced. As a result of the computed DOS plots, it appears that the electrical conductivity of the nanotubes may be modestly altered by mycolactone molecule adsorption. Despite this, the noncovalently functioned BNNTs retained the semiconductor features of the nanotubes. The adsorption of mycolactone on CNTs did not produce any additional states, and the HOMO and LUMO orbitals remained intact. The DOS profile of the CNTs appears to be unaltered by the non-covalent interaction of the mycolactone molecule, with the exception of slight alterations in peak strength in the lower areas. The un-separated HOMO and LUMO peaks of CNTs revealed that the CNTs retain their conducting properties even when a mycolactone molecule is adsorbed on them. The electrical conductivity of the BNNTs may be considerably modified by the mycolactone adsorption, while the conductance of the CNTs remains intact. This suggests that CNTs may be a more effective inhibitor of mycolactone than BNNTs.

### 3.2. Frontier Orbitals Analysis

**Figure 4:**
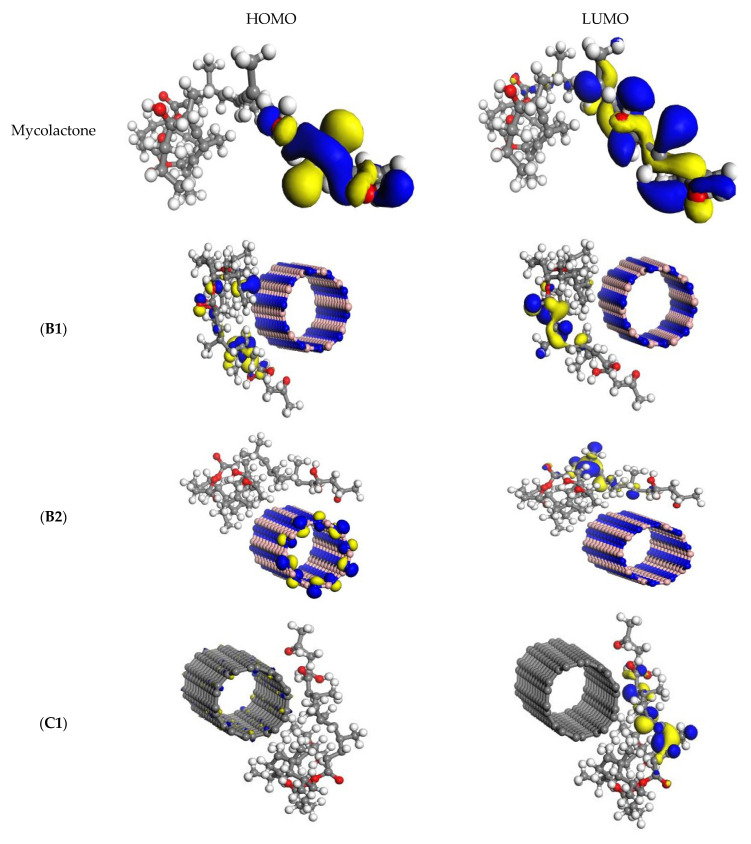

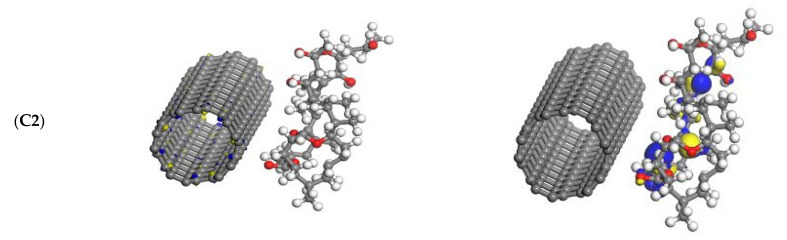
The frontier HOMO and LUMO orbitals of the mycolactone and its interaction with nanotubes. The isovalue of the isosurface is set as 0.03. (**B1**)—Mycolactone on a center of BNNT’s surface, (**B2**)—Mycolactone on the far end of the BNNT’s surface, (**C1**)—Mycolactone on the center of the CNT’s surface, and (**C2**)—Mycolactone on the far end of the CNT’s surface.

In the next stage, the frontier orbitals of the mycolactone molecule and nanotubes were studied to explore how interaction affects the electric charge properties of the nanotubes. In Figure 4, the isosurfaces of the mycolactone molecule’s frontier orbitals and the nanotubes that interact with it are shown. The isolated mycolactone’s HOMO and LUMO were both concentrated on the long chain ends, indicating that both orbitals were delocalized. The HOMO and LUMO of the non-covalently functionalized B1 were mostly found on the mycolactone molecule. In B2, the HOMO was mostly found on BNNT, while the LUMO was found on the mycolactone molecule. The electronic charges can then be transferred from the BNNT to the mycolactone molecule in the case of the B2 complex, but not in the case of the B1 complex. The HOMOs in mycolactone on CNTs were mostly found on the CNTs, while the LUMOs were mostly found on the mycolactone molecule. The atoms of mycolactone made no contributions to the border orbitals. Because CNTs are conductive, mycolactone’s electronic charges can easily be transferred within the CNTs rather than between the mycolactone and the CNTs. 

### 3.3. Chemical Reactivity Analysis

In order to investigate the effects of mycolactone on nanotube reactivity, the chemical potential (µ), global hardness (η), and electrophilicity index (ω) were calculated to study the reactivities of pristine and mycolactone-adsorbed nanotubes. Table 1 shows three DFT-based chemical descriptors for BNNTs and CNTs, respectively. In these quantities, µ is calculated as the average of HOMO and LUMO energies, η is calculated as the half of the differences between the HOMO and LUMO energies. Then, ω which is calculated according to the equation: ω = µ2/2η, can be used to estimate the reactivity of the molecule [38]. The electrophilic index of various chemical compounds and the rate of reaction in the biochemical system have been discovered to be related. When a chemical system receives additional charges from the environment, the electrical index influences its energy stability. The HOMO–LOMO gap was reduced by around 3 eV as a result of the mycolactone adsorption on BNNTs. The µ, η indexes were lowered by about 0.2–2 eV, while the ω index was increased by about 27 eV. When the mycolactone was adsorbed on the CNTs, no significant changes were observed for both the HOMO–LOMO gap and the three electrophilic indexes. It was then revealed that when the mycolactone molecule is adsorbed on the surface, the chemical stabilities of CNTs are maintained, while there is a large fluctuation in the reactivities of BNNTs. The 43.90 kcal/mol obtained was the maximum interaction energy between BNNTs and the mycolactone molecule, which is a bit low. However, −105.20 kcal/mol is the maximum interaction energy between CNTs and the mycolactone molecule, which makes CNTs more favorable than BNNTs.

### 3.4. Significance, Limitations, and Suggestions for Future Work

The precision of DFT for investigating biomolecular characteristics has been extensively studied. DFT has been shown to effectively predict the geometries of smaller organic compounds, indicating its efficacy in predicting the geometries of medicinal molecules. Various studies comparing DFT calculations and experimental values have been conducted, revealing the appropriateness of the DFT approach [39,40]. Other studies, such as charge transfer, prolonged pi conjugation, and bond cleavage [41,42] have pointed out some of its limits. 

SWNTs exhibited higher stability when adsorbed with a biomolecule than MWNTs [43], and when NT configuration (5,5) was compared to zig-zag and chiral configurations, the NT configuration (5,5) was found to be the most stable following biomolecular adsorption [25], so we may not expect the same behavior for MWNTs, zig-zag, and chiral configurations. For the remaining configurations, we advocate computational as well as experimental evaluations in the future. 

## 4. Conclusions

In conclusion, DFT calculations were used to investigate the ability of BNNTs and CNTs to act as mycolactone inhibitors in this study. As demonstrated by the optimized structure, the mycolactone molecule could adsorb parallel to the tube surface via π-π stacking interactions. The mycolactone primarily interacted with the nanotube surface via π-π stack interactions in parallel orientations. Because of these orientations, mycolactone’s ability to interact with nanotube surfaces via strong electrostatic interactions was limited. As a result, mycolactone’s interaction strength on the nanotube surface was weaker than in the end side case. The positive regions of the mycolactone surface cover a greater domain than the negative regions, indicating that the mycolactone was electrophilic rather than nucleophilic reactive. This indicated that the mycolactone molecule is more likely to bind to negatively charged surfaces. Because of the abundance of negative charges and aromaticity of the CNTs, the π-π stack interaction and electrostatic interaction of mycolactone on CNTs were stronger than on BNNTs, which explains the stronger interaction of mycolactone on CNTs. CNTs may thus be a more effective inhibitor of mycolactone toxin in biological systems. The CNTs’ unseparated HOMO and LUMO peaks revealed that the CNTs retain their conducting properties even when a mycolactone molecule was adsorbing on them. The mycolactone adsorption left the conductance of the CNTs unchanged, but the electrical conductivity of the BNNTs may be significantly altered. The CNTs could block the toxin without dramatically changing their characteristics in the process. This suggests that CNTs may be a better inhibitor of the mycolactone toxin. We anticipate that studying the adsorption and inhibitory capabilities of nanotubes will lead to the identification of new nanomedicine applications.

## Figures and Tables

**Table 1:** Computed adsorption energies (E_ads_), closest contact distance (R) between the binding site and the closest atom of the mycolactone molecule, HOMO, LUMO energies, band gap (E_gap_), chemical potential (µ), global hardness (η), and electrophilicity index (ω) for the most stable structures of the mycolactone-nanotube complexes. Units for Eads are kcal/mol, other energies in eV, and bond length in Å.

Systems	E_ads_	R	HOMO	LUMO	E_gap_	µ	η	ω
B1	−43.90	2.36	−5.01	−4.31	0.70	−4.66	0.35	31.02
B2	−36.40	2.48	−5.06	−4.23	0.83	−4.65	0.42	25.74
BNNT	-	-	−6.54	−2.10	4.44	−4.32	2.22	4.20
C1	−105.20	2.38	−3.86	−3.56	0.30	−3.70	0.16	44.10
C2	−96.77	2.74	−3.86	−3.55	0.31	−3.71	0.15	44.57
CNT	-	-	−3.83	−3.52	0.31	−3.67	0.16	42.94

## Data Availability

All data for this work are provided in the manuscript.

## Supplementary Materials

The following supporting information can be downloaded at: https://www.mdpi.com/article/10.3390/molecules27144440/s1.
