# Supplementary material for: Density Functional Theory-Based Studies Predict Carbon Nanotubes as Effective Mycolactone Inhibitors
Source: Molecules. 2022 Jul 11;27(14):4440. doi: 10.3390/molecules27144440 (PMC9316911; doi:10.3390/molecules27144440)
Supplement: Supplementary file 1 [file molecules-27-04440-s001.zip › Supplementary_File_1.pdf]

B1- ATOMIC POSITIONS in crystal coordinates for the Mycolactone on a center of BNNT’s surface structure used in the manuscript

B2- ATOMIC POSITIONS in crystal coordinates for the Mycolactone on a far end of BNNT’s surface structure used in the manuscript.

C1- ATOMIC POSITIONS in crystal coordinates for the Mycolactone on a center of CNT’s surface structure used in the manuscript.

C2- ATOMIC POSITIONS in crystal coordinates for the Mycolactone on a far end of CNT’s surface structure used in the manuscript.

BNNT (5, 5) - ATOMIC POSITIONS in crystal coordinates for the BNNT (5, 5) structures used in the manuscript

CNT (5,5) -ATOMIC POSITIONS in crystal coordinates for the CNT (5,5) structure used in the manuscript
